# Juvenile-Onset Diabetes and Congenital Cataract: “Double-Gene” Mutations Mimicking a Syndromic Diabetes Presentation

**DOI:** 10.3390/genes8110309

**Published:** 2017-11-07

**Authors:** Caroline Lenfant, Patrick Baz, Anne Degavre, Anne Philippi, Valérie Senée, Claire Vandiedonck, Céline Derbois, Marc Nicolino, Pierre Zalloua, Cécile Julier

**Affiliations:** 1 INSERM UMR-S 958, Faculté de Médecine Paris Diderot, University Paris 7 Denis-Diderot, Sorbonne Paris Cité, Paris 75010, France; caroline.lenfant@inserm.fr (C.L.); anne.degavre@inserm.fr (A.D.); anne.philippi@inserm.fr (A.P.); valerie.senee@inserm.fr (V.S.); claire.vandiedonck@inserm.fr (C.V.); 2 Department of Ophthalmology, Hôtel Dieu Hospital, Beirut 166830, Lebanon; patrickbaz57@yahoo.fr; 3 Centre National de Recherche en Génomique Humaine, Institut de Biologie François Jacob, Commissariat à l’Energie Atomique, Evry 91057, France; derbois@cng.fr; 4 Hôpital Femme-Mère-Enfant, Division of Pediatric Endocrinology, Hospices Civils de Lyon, Université Lyon 1, Bron cedex 69677, France; marc.nicolino@chu-lyon.fr; 5 School of Medicine, Lebanese American University, Beirut 1102 2801, Lebanon; pierre.zalloua@lau.edu.lb

**Keywords:** monogenic diabetes, congenital cataract, syndrome, whole exome sequencing, penetrance, consanguinity

## Abstract

Monogenic forms of diabetes may account for 1–5% of all cases of diabetes, and may occur in the context of syndromic presentations. We investigated the case of a girl affected by insulin-dependent diabetes, diagnosed at 6 years old, associated with congenital cataract. Her consanguineous parents and her four other siblings did not have diabetes or cataract, suggesting a recessive syndrome. Using whole exome sequencing of the affected proband, we identified a heterozygous p.R825Q *ABCC8* mutation, located at the exact same amino-acid position as the p.R825W recurring diabetes mutation, hence likely responsible for the diabetes condition, and a homozygous p.G71S mutation in *CRYBB1*, a gene known to be responsible for congenital cataract. Both mutations were predicted to be damaging and were absent or extremely rare in public databases. Unexpectedly, we found that the mother was also homozygous for the *CRYBB1* mutation, and both the mother and one unaffected sibling were heterozygous for the *ABCC8* mutation, suggesting incomplete penetrance of both mutations. Incomplete penetrance of *ABCC8* mutations is well documented, but this is the first report of an incomplete penetrance of a *CRYBB1* mutation, manifesting between susceptible subjects (unaffected mother vs. affected child) and to some extent within the patient herself, who had distinct cataract severities in both eyes. Our finding illustrates the importance of family studies to unmask the role of confounding factors such as double-gene mutations and incomplete penetrance that may mimic monogenic syndromes including in the case of strongly evocative family structure with consanguinity.

## 1. Introduction

Monogenic diabetes may account for 1–5% of all cases of diabetes [1,2], which is likely to be underestimated due to underdiagnosis. In current practice, monogenic diabetes is suspected in the case of diabetes with atypical presentation compared to common type 1 (T1D) and type 2 (T2D) diabetes, based on specific clinical or familial characteristics [3]. Most cases that are routinely screened for monogenic diabetes today present either with multi-generation transmission, suggesting Maturity-Onset Diabetes of the Young (MODY), or with extreme clinical presentation such as neonatal diabetes or syndromic features or both. Diabetes syndromes, where diabetes is associated with additional remarkable clinical or phenotypic features, is emerging as a new and highly heterogeneous group of diabetes, which may result from single mutations in various genes. Conversely, genetic studies of syndromic forms of diabetes have resulted in the identification of an increasing number of genes responsible for monogenic diabetes, that frequently have a recessive inheritance, including *WFS1* responsible for Wolfram syndrome [4], *EIF2AK3* responsible for Wolcott–Rallison syndrome [5] and *GLIS3* in neonatal diabetes and congenital hypothyroidism syndrome [6]. Here, we performed a genetic study of a girl born to consanguineous parents, affected by juvenile-onset insulin-treated diabetes associated with congenital cataract. In this case, the association of syndromic diabetes presentation in the child, parental consanguinity and healthy status of both parents and the four other siblings led to the suspicion of a new monogenic recessive syndrome. Using whole exome sequencing and genetic study of the entire family, we were able to determine the genetic origin of this syndromic presentation.

## 2. Patient, Family and Methods

### 2.1. Patient and Family

We studied a consanguineous Lebanese family, with one child affected by juvenile-onset diabetes and congenital cataract, that was recruited in the frame of a genetic study of juvenile-onset diabetes in Lebanon [7]. The four other children and the parents were healthy and were not known to have diabetes or cataract. The study was explained to the patient, the parents and three siblings, who agreed to participate in the genetic study and signed informed consents. One healthy sibling was not available for study. The study protocol was approved by the local ethics committee of the Chronic Care Center (Beirut, Lebanon) on 1 December 2001. Blood samples were obtained from the parents, the affected child and three healthy siblings. DNA was extracted using standard procedures.

### 2.2. Whole-Exome Sequencing

Exome sequencing was performed on the sequencing platform of the Institut de Génétique et de Biologie Moléculaire et Cellulaire (IGBMC, Illkirch, France). Exons of DNA blood samples were captured with In-solution Enrichment Methodology (SureSelectXT Target Enrichment System kit for Illumina Paired-End Sequencing Library version v1.5 November 2012 (Part Number G7530-90000); Agilent, Massy, France). The exon capture kit reference used was Human All Exon 50 Mb-ELID S02972011. Genomic DNA was sequenced using a HiSeq 2500 (Illumina, San Diego, CA, USA) as paired-end 100 base reads according to the manufacturer’s protocol. Image analysis and base calling were performed using CASAVA v1.8.2 (Illumina). Reads were mapped onto the reference genome hg19 using BWA v0.6.1 [8]. Variant discovery was done using GATK v2.5-2 [9] and Samtools v0.1.18 [10]. The overall sequencing coverage over the whole exome was 89% for a 10× depth of coverage, resulting in a mean sequencing depth of 77×. Exome variant analysis was then performed using an in-house python pipeline on genetic variation annotation results. Variants were filtered consecutively based on their quality (variant quality (Phred Q score) >20, genotype quality >20 and depth ≥ 5×), the predicted consequence on coding capacity (missense, nonsense, splice-site, and coding insertion/deletion—frameshift or inframe), and their rare status based on information available in public databases (Exome Aggregation Consortium (ExAC, release 0.3, 60,706 individuals) [11]; Exome Variant Server (EVS, release ESP6500SI-V2, 6503 individuals) and Single Nucleotide Polymorphism database (dbSNP, v.150)). In addition, data from 20 subjects sequenced on the same sequencing platform were used to filter platform-specific artifacts. Variants that were found with a Minor allele frequency (MAF) >0.005 in these public databases were excluded. Duplicate filtering of exome sequencing results of the patient was performed: (1) for variants found in the homozygous status and (2) for variants found in known monogenic diabetes genes in the homozygous or heterozygous status. Additional relevant information automatically collected from public databases by the in-house analysis pipeline was then considered for further selection of variants and genes of interest, including annotations extracted from NCBI (ClinVar, Omim, MedGen, dbSNP clinical significance and PubMed search using patient-specific keywords). Allele frequencies of selected variants were then further investigated in additional databases: the Genome Aggregation Database, an extension of ExAC (gnomAD, 138,632 individuals), and the Greater Middle East Variome Project (GME, 2497 individuals) [12], and sub-populations within these cohorts.

### 2.3. Mutation Confirmation and Genotyping in the Whole Family

Variants in the *ABCC8* and *CRYBB1* genes identified by exome sequencing in the patient were confirmed and genotyped in the whole family using Sanger sequencing on Polymerase Chain Reaction (PCR)-amplified DNA. PCR amplification and sequencing primers are shown in Supplementary Table S1. 

### 2.4. HLA Typing

HLA-DRB1*03 (DR3) and -DRB1*04 (DR4) typing was performed by TaqMan assay using the tagging SNPs rs2187668 and rs7454108 respectively [13,14].

### 2.5. Protein Structural Modeling

The X-ray crystal structure of human CRYBB1 (Gly71, WT) from the Protein Data Bank (PDB: 1OKI) was used as reference [15]. The mutated CRYBB1 (Ser71, mutated) was modeled based on 1OKI structure using MODELLER 9.11 package [16] to satisfy the spatial constraints of the two monomers (A and B chains). Intra- and inter-protein chain interactions were calculated using the Protein Interaction Calculator (PIC) server on the wild type (WT) and mutated structures [17] using default parameters. Specifically, for hydrophobic interactions, residues Ala, Val, Leu, Ile, Met, Phe, Trp, Pro, Tyr were considered as interacting if they fell within 5 Å of each other; for hydrogen bond definition, a donor–acceptor distance cutoff of 3.5 Å was applied; for aromatic interaction, pairs of phenyl ring centroids were separated by a distance of 4.5 to 7 Å; Cation–Pi interactions were defined by a cationic chain (Lys or Arg) within 6 Å of an aromatic side chain (Phe, Tyr or Trp). The figure was generated using RasMol v2.7.5.2 [18].

## 3. Results

### 3.1. Description of an Apparently New Syndrome with Juvenile-Onset Diabetes and Congenital Cataract

The index case (subject 6) was diagnosed with diabetes at the age of 6 years, with symptoms of polyuria, polydipsia and weight loss. She has been treated by insulin injection continuously since the onset of diabetes and until the time of the study at age 19 years. Islet-specific autoantibodies and C-peptide were not measured at disease onset or later. In addition, she had congenital cataract, which was severe in the right eye and operated at the age of 18 months, and was limited to lens opacity in the left eye, not operated. She was first seen at the clinic at the age of 11 years. In the right eye, she had complete loss of visual perception, high intraocular pressure, constant exotropia, dystrophic corneal opacity and optic atrophy. In the left eye, visual acuity was 5/10 with no correction, due to anterior-cortical opacity of the lens, while there was no optic atrophy; the left eye was fixating in adduction. At the time of the study, her clinical status remained stable, and in particular there was no optic atrophy of the left eye. The patient did not have retinopathy, hearing loss, diabetes insipidus, neurologic problems, nephropathy or hypertension. She had normal development and intellectual ability. Diabetes was well controlled with relatively low insulin doses (mean HbA1c of 7.5% on insulin doses of 0.7 IU/kg/day on average). Her body mass index (BMI) ranged between 25 and 27. With the association of juvenile-onset diabetes and optic atrophy, the clinical presentation showed similarities with Wolfram syndrome, but the absence of optic atrophy in the other eye and the absence of aggravation over years, as well as the absence of hearing loss, diabetes insipidus, renal and neurologic features did not support this diagnosis. In our patient’s case, unilateral optic atrophy appeared as a likely consequence of long-term ocular hypertonia of the eye that had been operated for congenital cataract. The association of juvenile-onset diabetes and congenital cataract was unique and in particular it was the only case found among a total of 400 Lebanese juvenile onset diabetes families studied [7]. Parents were second cousin consanguineous and were not known to be diabetic at the time of the study, based on their self-reports, as were the four other siblings (aged 12 to 28 years old). The association of juvenile-onset diabetes and congenital cataract in a child born to healthy consanguineous parents with no other family member affected by either diabetes or congenital cataract suggested the hypothesis of a new monogenic recessive syndrome. 

### 3.2. Identification of a Heterozygous Mutation in the ABCC8 Gene Likely Responsible for Juvenile-Onset Diabetes

In order to identify the putative causative gene and mutation responsible for the syndromic presentation of the index case, we performed exome sequencing of the patient. We conducted two independent analyses: (1) selection of rare variants from known monogenic diabetes genes present in the patient in the causative diabetes genetic status (homozygous or heterozygous status or both depending on the gene); and (2) selection of all rare coding variants homozygous in the patient. We did not identify any rare homozygous variant in any known monogenic diabetes gene, but we identified a rare heterozygous variant in the *ABCC8* gene, encoding sulphonylurea receptor 1 (SUR1, Table 1), which is a component of the ATP-sensitive potassium (K_ATP_) channels that plays a key role in the regulation of insulin secretion by pancreatic β–cells. Activating mutations in *ABCC8* are known to cause neonatal diabetes, maturity onset diabetes of the young (MODY) and T2D, while loss of function mutations lead to an opposite phenotype with hyperinsulinism and hypoglycemia [19]. The variant identified (chr11:g.17434942G>A, hg19) results in non-synonymous substitution of an arginine by a glutamine at position 825 (p.R825Q) and is absent from ExAC/gnomAD (138,632 subjects) and GME (2497 subjects) databases, and observed in only one subject from EVS, in the heterozygous status (MAF = 1/12,986 or 0.000077). The estimated allele frequency across all these independent cohorts is 3.4 × 10^−6^ (Table 1). Genotyping of the variant in the whole family confirmed the heterozygous status of the diabetic proband, as well as her mother and her brother who were not known to be diabetic at the time of the study, while all other non-diabetic family members were homozygous for the reference allele. The R825 is located in the first nucleotide-binding domain (NBD1) within the ABC transport domain of SUR1, a highly conserved region that associates in a heterodimer with NBD2 to form a catalytic ATP-binding site that is involved in binding and hydrolysis of Mg-ATP to regulate the K_ATP_ channel [20]. This variant is predicted to be deleterious based on 9 out of 11 in silico pathogenicity programs from ANNOVAR (Table 1, Supplementary Table S2). Noteworthy, the replacement of arginine by tryptophan at exactly the same position (p.R825W) has previously been reported in the heterozygous status in subjects with permanent neonatal diabetes (PNDM), transient neonatal diabetes (TNDM), relapsed TNDM, adult-onset T2D as well as in non-diabetic subjects, and in the homozygous or compound heterozygous status in patients with PNDM [20,21,22,23,24,25,26]. De Wet et al. [20] performed detailed functional studies of the R825W mutation and showed that this mutation resulted in slowing down the deactivation of K_ATP_ channel activity and increasing its resting activity, thereby resulting in reduced insulin secretion and leading to diabetes. These observations strongly suggest that the ABCC8-p.R825Q variant, affecting the same residue, is pathogenic and responsible for diabetes in the index patient. In the absence of autoantibody data, we cannot however formally exclude the possibility of autoimmune T1D. The fact that diabetes was relatively well controlled with low insulin doses when BMI was in the high range further argues for a causative *ABCC8* mutation. Indeed, in T1D patients, the mean insulin dose is generally increased in overweight subjects due to insulin resistance. Furthermore, the absence of any other autoimmune disorder such as thyroiditis or coeliac disease that are frequently associated with T1D, either in the patient or in other family members, also agrees with this hypothesis. 

Remarkably, HLA typing indicated that the patient was HLA-DR3/3, hence a carrier of a T1D risk genotype [27], while both parents were HLA-DR3 heterozygous and all the unaffected siblings were HLA-DR3 negative, which may rather support the possible contribution of an autoimmune component.

### 3.3. Identification of a Homozygous Mutation in the CRYBB1 Gene Responsible for Congenital Cataract

**Figure 1:**
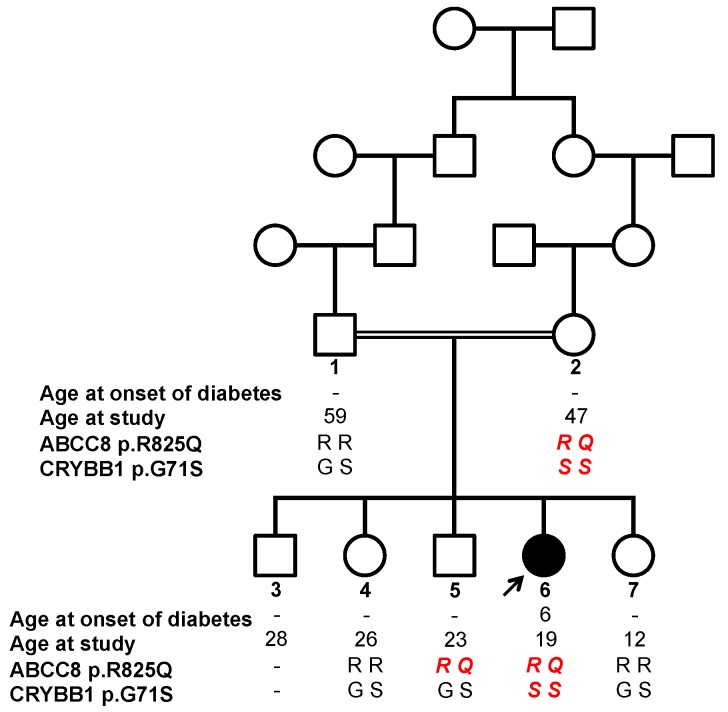
*ABCC8* and *CRYBB1* mutations in the family. Genotypes of the *ABCC8* and *CRYBB1* mutations (protein changes) identified by whole exome sequencing in the patient (subject 6, arrow) are shown in the whole family. Pathogenic genotypes for each gene are shown in bold red italics. Filled symbols: diabetes and cataract, empty symbols: no known diabetes and no cataract. Subject 3 was not genetically studied.

**Figure 2:**
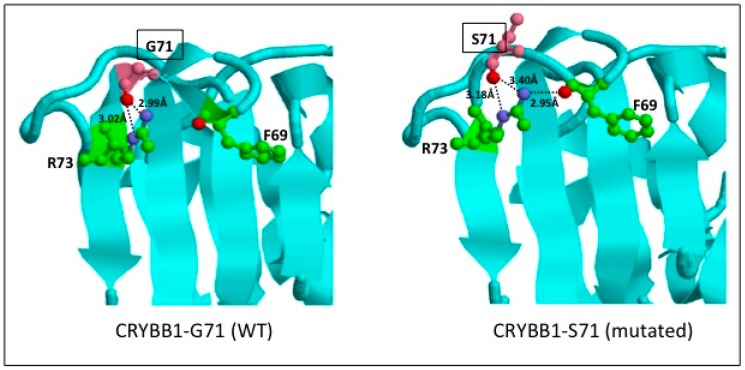
Structure of a CRYBB1 monomer and predicted consequences of the CRYBB1 p.Gly71Ser mutation (left: wild type (WT), right: mutated). CRYBB1 structure (WT) is according to Protein Data Base (PDB) (1OKI), and corresponds to residues 54–236 of the protein, with 1.4 Å of resolution. Amino acid residues are numbered according to the CRYBB1 UniProtKB sequence (p53674). In the mutated model (CRYBB1 Ser71), a new H-bond interaction was predicted between oxygen atom (red) of Phe69 (green) and azote atom (purple) of Ser71 (pink) residues, as well as the loss of part of the β strand. For convenience, only one chain of the CRYBB1 homodimer is represented. H-bonds are shown in black dotted lines.

We identified 25 rare homozygous coding variants after filtering using our in-house pipeline (MAF <0.005 in ExAC, EVS and dbSNP; Supplementary Table S3). Subsequent filtering in all population subgroups from gnomAD, EVS and GME (maximum MAF <0.005), reduced it to 13 variants in 13 genes. Among these, *CRYBB1*, encoding β-crystallin B1, appeared as a major candidate gene and the only one for congenital cataract in the patient. Crystallins are the main structural components of the vertebrate eye lens [28]. Mutations in *CRYBB1* and in other crystallin genes represent a large proportion of causative congenital cataract mutations discovered to date, most of which are dominant and some recessive [29]. The *CRYBB1* variant identified (chr22:g.27008124G>A, hg19) results in a non-synonymous substitution (p.G71S). It is absent from all public databases (ExAC/gnomAD, EVS, GME; a total of 147,632 subjects sequenced) and has never been reported before (Table 1). Genotyping of this variant in the entire family showed that both the mother (unaffected) and the affected proband are homozygous for the mutation while the father and other siblings (all unaffected) are heterozygous, compatible with a recessive mutation with incomplete penetrance (Figure 1). CRYBB1 and other β- and γ-crystallins are structurally conserved proteins, composed of four Greek key motifs organized in two domains (N- and C-terminal). Each Greek key domain is composed of four β-strands that are precisely folded into a tridimensional structure. The CRYBB1-p.G71S mutation affects a highly conserved glycine located in the β2-strand within the Greek key domain 1 [15]. This mutation is predicted to be damaging to protein function by all 11 programs tested (Table 1, Supplementary Table S2). Tridimensional modelling of the homodimeric wild type and mutated protein predicted conformational changes, including the gain of a H-bond interaction with F69 residue and the loss of a part of the β2 strand secondary structure, further supporting a functional impact of the mutation (Figure 2). Previous structural studies have highlighted the role of this specific glycine residue, which is conserved across all the Greek key domains of β- and γ-crystallins, to insure the proper protein folding and stability [28,30]. Interestingly, a mutation in the crystallin γS (CRYGS) at the homologous glycine p.G18V (see ref [28] for protein alignments) also causes cataract [31]. Consistently, this latter mutation has been shown to affect protein folding by decreasing its thermal stability and solubility [32,33], to increase the aggregation propensity of the protein [33] and to increase hydrophobic surface exposure [34]. These observations strongly suggest that the homozygous CRYBB1-p.G71S mutation is responsible for the congenital cataract of the patient, with incomplete penetrance noted in the unaffected mother. Overall, we conclude that the syndromic diabetes presentation of the index case results from the combination of a heterozygous ABCC8-p.R825Q mutation causing diabetes and a homozygous CRYBB1-p.G71S mutation causing cataract.

## 4. Discussion

The association of juvenile-onset insulin-treated diabetes with congenital cataract in a single affected child among five siblings born to healthy consanguineous parents initially prompted us to consider a monogenic inheritance of a new recessive syndrome with diabetes and congenital cataract as a plausible hypothesis. Our detailed genetic and familial study revealed that this disease association was rather explained by independent mutations in two genes that were known to cause diabetes (*ABCC8*) and congenital cataract (*CRYBB1*), respectively dominant and recessive, both of which with incomplete penetrance. Remarkably, the mother, who was healthy at the time of the study, shared the same disease-causing genotypes at both genes and was most likely inbred herself. In addition, the patient carried a HLA risk genotype for T1D, which is also relatively common in the general population, and in the absence of autoantibody data we cannot exclude that the patient may have an autoimmune component to her diabetes. The clinical presentation of the patient shares features of T1D (juvenile onset diabetes with acute onset and continuous insulin-treatment) and of *ABCC8*-mutated diabetes, including good control with relatively low insulin doses and no history of associated autoimmune disorders.

The family study showed that both the mother and one sister, who were not known to be diabetic at the time of the study at ages 47 years and 23 years respectively, were heterozygous for the same *ABCC8* mutation as the affected child who had diabetes at the age of 6 years old. It should be noted, however, that diabetes assessment of the relatives was self-reported and could not be re-evaluated following the genetic study, which does not exclude the possibility of pre- or mild-diabetes status in these mutation carriers. In any case, the early and severe diabetes status of the affected child contrasts with the reported healthy status of the heterozygous mother and sibling. Incomplete penetrance and variable expressivity of *ABCC8* mutations is well documented. In particular, a previous report showed that the an *ABCC8* mutation segregating in a 3-generation family led to neonatal diabetes in one child, asymptomatic T2D in the father and asymptomatic impaired fasting glucose in the grand-mother [35]. Noteworthy, the ABCC8-p.R825W mutation, affecting precisely the same amino-acid as the patient’s ABCC8-p.R825Q, has been associated with a very variable diabetes expressivity, ranging from neonatal diabetes to adult-onset diabetes and non-diabetic status. Mutations in *ABCC8* and in other monogenic genes are now emerging as a significant cause of juvenile-onset diabetes [36,37]. In the present case, the additional HLA risk carried by the patient may have precipitated diabetes at a young age, compared to the mother and sister *ABCC8* carriers who did not have the additional HLA risk. Remarkably, the presence of T1D-specific autoantibodies has been detected in diabetic patients carrying *ABCC8* mutations [23]. It is conceivable that autoimmune and non-autoimmune mechanisms may synergize to result in insulin-dependent juvenile-onset diabetes, as we previously described in the case of a *ALMS1* mutated patient carrying an independent HLA risk and presenting with insulin-dependent diabetes [38].

The family study also revealed that the mother was homozygous for the same *CRYBB1* mutation as the child, despite having no cataract at the age of 47 years. This case is remarkable since incomplete penetrance noticed in the mother also manifests to some extent in the child herself, who had severe congenital cataract in one eye but only partial opacity of the other eye, that did not significantly evolve in 8 years of follow-up. Therefore, incomplete penetrance of this *CRYBB1* mutation manifests here at the level of the whole subject (affected vs. unaffected subjects with the disease-causing genotype) and between the two eyes of the patient (severe vs. mild cataract). Evidence for incomplete penetrance of congenital cataract has been previously reported for a mutation in the *GJA8* gene [39], and this is to our knowledge the first case of an incomplete penetrance for a *CRYBB1* mutation. *CRYBB1* mutations may be dominant or recessive [40]. Our family observation strongly suggests that the CRYBB1-p.G71S mutation is recessive, consistent with the recessive inheritance of the close p.E75K and p.D85N mutations [40] located in the same Greek key domain. 

In addition to juvenile-onset diabetes and neonatal cataract, the patient also had optic atrophy in the most severely affected eye, most likely as a consequence of ocular hypertonia, resulting in some similitude with Wolfram syndrome. Although, in this case, the genetic and family study unambiguously ruled out a monogenic syndrome, it is interesting that a new monogenic syndrome associating neonatal/infancy onset diabetes, congenital sensorineural deafness and congenital cataract has very recently been described in patients carrying heterozygous severe *WFS1* mutations [41], showing some similitude with the clinical presentation of the index case. Cataracts have also been reported in Wolfram patients carrying biallelic *WFS1* mutations, although this is not a feature of this syndrome, and dominant isolated congenital cataract has been described in members from an extended family carrying a specific heterozygous *WFS1* mutation [42]. In the present case, the index patient did not carry any rare homozygous or heterozygous mutation in the *WFS1* gene, and the two observations appear to be coincidental.

Overall, our findings support that the syndromic presentation of the index case is caused by the combination of independent mutations in the *ABCC8* and *CRYBB1* genes. In the absence of extensive immunological and metabolic phenotyping of the patient and of heterozygous *ABCC8* mutation carriers, we cannot however exclude the possibility of an autoimmune component to diabetes. 

Our observation highlights the importance of family studies to dissect the origin of clinical traits and particularly rare syndromes, where additional factors such as multiple genetic and non-genetic factors, incomplete penetrance as well as known or unknown consanguinity may result in unexpected situations. 

## Figures and Tables

**Table 1:** Characteristics of the mutations identified in the *ABCC8* and *CRYBB1* genes.

Chromosome Position (hg19)	Gene	dbSNP rsID	cDNA		Protein		Allele Counts WT/Mutated (MAF) in Public Databases			Estimated MAF *	In silico Pathogenicity Based on Annovar Prediction Programs
			Refseq	Nucleotide Change	Refseq	Amino Acid Change	gnomAD (*N* = 138,632)	EVS (*N* = 6503)	GME (*N* = 2497)	gnomAD, EVS, GME	
chr11: g.17434942G>A	*ABCC8*	rs375172221	NM_000352	c.2474G>A	NP_000343	p.Arg825Gln	246,266/0	12,985/1 (0.000077)	1986/0	3.4 × 10^-6^	deleterious (9/11)
chr22: g.27008124G>A	*CRYBB1*	NA	NM_001887	c.211G>A	NP_001878	p.Gly71Ser	Absent	Absent	Absent	Absent	deleterious (11/11)

* Estimated Minor allele frequency (MAF) was based on genotyped subjects of all independent cohorts using allele counts at the specific position. In silico pathogenicity shown is the count of deleterious predictions based on 11 options of nine different prediction programs assembled by ANNOVAR (Supplementary Table S2). WT: wild type; *N*: size of the cohorts (number of subjects); cDNA: complementary DNA; gnomAD: Genome Aggregation Database; EVS: Exome Variant Server; GME: Greater Middle East Variom Project.

## Supplementary Materials

The following are available online at www.mdpi.com/2073-4425/8/11/309/s1. Table S1: PCR amplification and sequencing primers used for *ABCC8* and *CRYBB1* mutations genotyping; Table S2: Predicted consequences of *ABCC8* and *CRYBB1* mutations according to ANNOVAR prediction programs; Table S3: Rare homozygous variants identified by whole exome sequencing of the patient.
