# Supplementary material for: Juvenile-Onset Diabetes and Congenital Cataract: “Double-Gene” Mutations Mimicking a Syndromic Diabetes Presentation
Source: Genes (Basel). 2017 Nov 7;8(11):309. doi: 10.3390/genes8110309 (PMC5704222; doi:10.3390/genes8110309)
Supplement: Supplementary file 1 [file genes-08-00309-s001.pdf]

SUPPLEMENTARY MATERIALS

Juvenile-onset diabetes and congenital cataract: « double-gene » mutations mimicking a syndromic diabetes presentation

Supplementary Table S1. PCR amplification and sequencing primers used for *ABCC8* and *CRYBB1* mutations genotyping

Amplified Region	Map Position*			PCR amplification and sequencing primers (5'-3')		Product size (bp)
	Chr	Start	End	Forward	Reverse	
ABCC8 exon 20	11	17434647	17435217	AAACGCTGTGTGAAGTGCTG	ACAATACAACCCCAGGCTGA	571
CRYBB1 exon 3	22	27007762	27008261	AAGCAGCATTTCTCCAGAGC	TCACAAACTGTGGCTCATCAC	520

* Mapping position on hg19. Chr: chromosome; bp : base pairs.

Supplementary Table S2. Predicted consequences of *ABCC8* and *CRYBB1* mutations according to Annovar prediction programs

Prediction programs		ABCC8 – p.R826Q		CRYBB1 – p.G71S	
		Score	Prediction	Score	Prediction
SIFT		0.01	Deleterious	0.0	Deleterious
Polyphen 2	HDIV	1.0	Damaging	1.0	Damaging
	HVAR	0.997	Damaging	1.0	Damaging
LRT		0.0	Deleterious	0.0	Deleterious
Mutation Taster		1.0	Disease causing	1.0	Disease causing
Mutation Assessor		1.115	Low	3.985	High
FTHMM		-1.39	Tolerated	-5.28	Deleterious
Meta	SVM	0.230	Deleterious	1.040	Deleterious
	LR	0.585	Deleterious	0.980	Deleterious
VEST3		0.663	Deleterious*	0.861	Deleterious**
CADD	Phred	33	Deleterious	35	Deleterious

*p-value = 0.003, **p-value = 0.01

In silico prediction of the impact and severity of mutations on protein function was performed using Annovar's prediction programs and ljb26_all database [1]. This database is created based on the dbNSFP database v2.6 [2,3] which is a database of human non synonymous SNV and their functional predictions and annotations. A total of 11 options from 9 independent programs called by Annovar are shown, with the scores, predictions and associated statistics specific to these programs.

Supplementary Table S3. Rare homozygous variants identified by whole exome sequencing of the patient

Chrom	Map position	Ref	Alt	rs	Gene	Transcript	cDNA	Protein	MAF EVS	MAF gnomAD	MAF GME	Max MAF (gnomAD, EVS,GME)	Population
chr1	1309567	G	A	rs199844974	*AURKAIP1*	NM_001127230	c.C311T	p.P104L	0	0.0003	0.0005	0.0040	gnomAD(EAS)
chr1	47606528	G	C	.	*CYP4A22*	NM_001010969	c.G272C	p.W91S	0	0	0	0	None
chr1	62916290	A	G	rs141001844	*USP1*	NM_001017415	c.A1996G	p.I666V	0.0013	0.0029	0.0211	0.0526	GME(AP)
chr1	186330768	T	C	rs201760745	*TPR*	NM_003292	c.A944G	p.K315R	0.0002	0.0003	0.0040	0.0117	GME(AP)
chr3	38292941	G	A	rs145431865	*OXSR1*	NM_005109	c.G1423A	p.D475N	0.001	0.0007	0.0035	0.0500	GME(Israel)
chr4	110384624	T	C	rs192938684	*SEC24B*	NM_006323	c.T701C	p.I234T	7.7E-05	0.0003	0.0005	0.0027	GME(NEA)
chr4	110763634	A	G	rs142582141	*RRH*	NM_006583	c.A730G	p.I244V	7.7E-05	4.1E-05	0	0.0001	EVS(EA)
chr4	110763635	T	G	rs202014616	*RRH*	NM_006583	c.T731G	p.I244S	0	0.0002	0.0030	0.0054	GME(NEA)
chr10	50533627	G	C	.	*C10orf71*	NM_001135196	c.G3037C	p.D1013H	0	1.3E-05	0.0010	0.0263	GME(SD)
chr10	52569773	G	C	.	*A1CF*	NM_001198820	c.C1514G	p.A505G	0	0	0.0005	0.0031	GME(TP)
chr12	102590058	T	A	.	*PARPBP*	NM_017915	c.T1729A	p.F577I	0	9.7E-05	0	0.0008	gnomAD(SAS)
chr12	104147024	C	T	rs150749035	*STAB2*	NM_017564	c.C6607T	p.R2203C	7.7E-05	0.0006	0.0005	0.0046	gnomAD(SAS)
chr15	57355983	A	G	.	*TCF12*	NM_003205	c.A184G	p.T62A	0	0	0	0	None
chr15	79058757	C	T	.	*ADAMTS7*	NM_014272	c.G3496A	p.A1166T	0	4.3E-05	0	0.0002	gnomAD(Other)
chr16	88599697	A	C	.	*ZFPM1*	NM_153813	c.A1331C	p.E444A	0	0.0002	0	0.0003	gnomAD(SAS)
chr17	2595308	T	C	.	*CLUH*	NM_015229	c.A3527G	p.Y1176C	0	4.4E-06	0.0041	0.0179	GME(AP)
chr17	3301139	A	C	rs148878494	*OR1E1*	NM_003553	c.T566G	p.F189C	0	0.0034	0.0042	0.0194	gnomAD(EAS)
chr17	3977476	G	A	.	*ZZEF1*	NM_015113	c.C3653T	p.A1218V	0	0	0	0	None
chr17	4837131	C	T	rs139921368	*GP1BA*	NM_000173	c.C1232T	p.P411L	0.0003	0.0009	0.0060	0.0234	GME(AP)
chr17	4907312	C	T	rs145650252	*KIF1C*	NM_006612	c.C884T	p.S295L	0.0003	0.0004	0.0010	0.0086	GME(SD)
chr17	5984001	T	C	rs199969111	*WSCD1*	NM_015253	c.T23C	p.L8P	0.0005	0.0013	0.0060	0.0152	GME(CA)
chr17	6716320	C	G	rs150419358	*TEKT1*	NM_053285	c.G682C	p.A228P	0.0003	0.0001	0.0005	0.0006	gnomAD(EAS)
chr20	23350290	G	A	rs144872945	*GZF1*	NM_001317019	c.G1697A	p.R566H	0.001	0.0006	0.0050	0.0109	GME(NEA)
chr20	29977000	A	C	rs149638804	*DEFB119*	NM_153323	c.T95G	p.L32W	0.0008	0.0014	0.0086	0.0146	GME(AP)
**chr22**	**27008124**	**C**	**T**	**.**	***CRYBB1***	**NM_001887**	**c.G211A**	**p.G71S**	**0**	**0**	**0**	**0**	**None**

Variants that were predicted to affect the coding capacity of proteins and whose minor allele frequency (MAF) was <0.005 in Exome Variant Server (EVS), Exome Aggregaiton Consortium (ExAC) and dbSNP databases were selected (N=25). For these variants, allele frequencies in EVS, Genome Aggregation Database (gnomAD) and Greater Middle East Variome Project (GME) are shown, and the maximum MAF in sub-populations (Max MAF) within these. EVS population: EA: European American; GnomAD populations: EAS: East Asian, SAS: South Asian; GME populations: AP: Arabian Peninsula, NEA: North-East Africa, SD: Syrian Desert, TP: Turkish Peninsula, CA: Central Asia. Maximum number of subjects sequenced in the consortium cohorts: EVS: 6503; gnomAD: 138632; GME: 2497. 13 of these variants had a Max MAF <0.005 in all sub-populations (lines shown in clear background): *AURKAIP1*, *CYP4A22*, *SEC24B*, *RRH*, *A1CF*, *PARPBP*, *STAB2*, *TCF12*, *ADAMTS7*, *ZFPM1*, *ZZEF1*, *TEKT1* and *CRYBB1*. A unique variant was located in a gene known to be causative of congenital cataract (*CRYBB1* gene, bolded, MAF=0 in all populations), and was therefore selected for this study. Ref : reference allele ; Alt : alternative allele.

References

1. Wang, K.; Li, M.; Hakonarson, H. Annovar: Functional annotation of genetic variants from high-throughput sequencing data. *Nucleic Acids Res.* 2010, *38*, e164.
2. Liu, X.; Jian, X.; Boerwinkle, E. Dbnsfp v2.0: A database of human non-synonymous snvs and their functional predictions and annotations. *Hum Mutat.* 2013, *34*, E2393-2402.
3. Kircher, M.; Witten, D.M.; Jain, P.; O'Roak, B.J.; Cooper, G.M.; Shendure, J. A general framework for estimating the relative pathogenicity of human genetic variants. *Nat Genet.* 2014, *46*, 310-315.
